# Chromosomal Aberrations in Bladder Cancer: Fresh versus Formalin Fixed Paraffin Embedded Tissue and Targeted FISH versus Wide Microarray-Based CGH Analysis

**DOI:** 10.1371/journal.pone.0024237

**Published:** 2011-09-01

**Authors:** Elena Panzeri, Donatella Conconi, Laura Antolini, Serena Redaelli, Maria Grazia Valsecchi, Giorgio Bovo, Francesco Pallotti, Paolo Viganò, Guido Strada, Leda Dalprà, Angela Bentivegna

**Affiliations:** 1 Department of Neuroscience and Biomedical Technologies, University of Milan-Bicocca, Monza, Italy; 2 Medical Genetics Laboratory, S. Gerardo Hospital, Monza, Italy; 3 Department of Clinical Medicine and Prevention, Center of Biostatistics for Clinical Epidemiology, University of Milan-Bicocca, Monza, Italy; 4 Department of Pathology, S. Gerardo Hospital, Monza, Italy; 5 Department of Pathology, Fondazione IRCCS Cà Granda, Ospedale Maggiore Policlinico, Milano, Italy; 6 Urology Division, Bassini ICP Hospital, Milano, Italy; Health Canada, Canada

## Abstract

Bladder carcinogenesis is believed to follow two alternative pathways driven by the loss of chromosome 9 and the gain of chromosome 7, albeit other nonrandom copy number alterations (CNAs) were identified. However, confirmation studies are needed since many aspects of this model remain unclear and considerable heterogeneity among cases has emerged. One of the purposes of this study was to evaluate the performance of a targeted test (UroVysion assay) widely used for the detection of Transitional Cell Carcinoma (TCC) of the bladder, in two different types of material derived from the same tumor. We compared the results of UroVysion test performed on Freshly Isolated interphasic Nuclei (FIN) and on Formalin Fixed Paraffin Embedded (FFPE) tissues from 22 TCCs and we didn't find substantial differences. A second goal was to assess the concordance between array-CGH profiles and the targeted chromosomal profiles of UroVysion assay on an additional set of 10 TCCs, in order to evaluate whether UroVysion is an adequately sensitive method for the identification of selected aneuploidies and nonrandom CNAs in TCCs. Our results confirmed the importance of global genomic screening methods, that is array based CGH, to comprehensively determine the genomic profiles of large series of TCCs tumors. However, this technique has yet some limitations, such as not being able to detect low level mosaicism, or not detecting any change in the number of copies for a kind of compensatory effect due to the presence of high cellular heterogeneity. Thus, it is still advisable to use complementary techniques such as array-CGH and FISH, as the former is able to detect alterations at the genome level not excluding any chromosome, but the latter is able to maintain the individual data at the level of single cells, even if it focuses on few genomic regions.

## Introduction

Bladder cancer is the seventh most common cancer worldwide [1] and the fourth most common cancer diagnosed in men in the USA and European countries [2]. Transitional Cell Carcinoma (TCC) comprises the majority of bladder cancers accounting for more than 90%. At presentation, the majority (∼70%) are superficial, exophytic, papillary tumors that are well-differentiated (low-grade, LG) and do not penetrate the epithelial basement membrane (stage Ta) [1], [3]; the remaining are muscle invasive (T2–T4) or microinvasive tumors (T1), that have penetrated the lamina propria but are not invading the muscle. In this minority, the tumor epithelium is poorly differentiated (high-grade, HG) and often associated with carcinoma *in situ* (CIS), which despite its superficiality is composed of poorly differentiated epithelium. This is thought to be representative of a precursor lesion [1]. Prognosis for LG Ta tumors is generally good because such tumors rarely progress, but monitoring is necessary given the significant risk of recurrence (up to 70%) [4]; this is necessary also for HG Ta (TaG3) and T1 tumors that represent a high risk of progression to muscle invasion. For patients with muscle invasive tumors (≥T2), metastasis is a major clinical problem and cystectomies are usually indicated. Prognosis is relatively poor with only 50% survival at 5 years since diagnosis [4].

The biological differences among these groups probably reflects the underlying genetic heterogeneity which leads to specific pathways of tumor development and progression. Innumerable studies have traced the status of known oncogenes and tumor suppressor genes and have revealed several recurring chromosomal changes associated with the pathologic stage and/or outcome of the tumor [5], [6]. Moreover, based on the well known genetic alterations of bladder cancer, a multi-target fluorescence in situ hybridization (FISH) assay has been developed [7]. The UroVysion FISH detection system, approved by the U.S. Food and Drug Administration, is based on three centromeric probes for chromosomes 3, 7 and 17 and a fourth probe to the 9p21 region, for the detection of chromosomal aneusomy and/or deletion of 9p21 locus, which are common genetic alterations in TCCs [8], [9]. UroVysion has been initially used in the last decade only for the surveillance, but recently also as a bladder cancer screening tool in patients with hematuria [10]–[12]. However, other methods have been applied to detect copy number changes associated with tumor development and progression of TCC. Conventional comparative genomic hybridization (CGH) studies have provided a great deal of information, including the identification of a number of genomic regions of DNA amplification containing known or candidate oncogenes [13]–[15]. On the other hand, the location of tumor suppressor genes in TCC have largely been identified by loss of heterozygosity (LOH) analysis [16]. With the use of the high-resolution mapping of array-based CGH, novel copy number alterations (CNAs) were identified in many small genomic regions that were not detected in previous studies [17]–[19]. The data collected so far, in addition to the identification of at least two cytogenetic pathways for tumor development, i.e. the loss of chromosome 9 and the gain of chromosome 7 [20]–[22], may be helpful in designing new individualized therapies. However confirmation studies are needed since many aspects of this model remain unclear, in particular on the chronological order of the aberrations during the disease progression. For a sensible identification of the genes underlying the chromosomal abnormalities, it becomes crucial to use reliable techniques and to go through the data validation process. This issue was recently addressed for prostate and breast cancer, gliomas and multiple myeloma [23]–[27], but not for bladder cancer. Although Formalin Fixed Paraffin Embedded (FFPE) specimens has several advantages, such as the certainty of histological diagnosis and allow retrospective studies of a large number of samples, fresh tissues are considered the most reliable for molecular genetic analysis; they provide a comprehensive analysis of the biopsy, although the material does not have a histological diagnosis.

**Figure 1:**
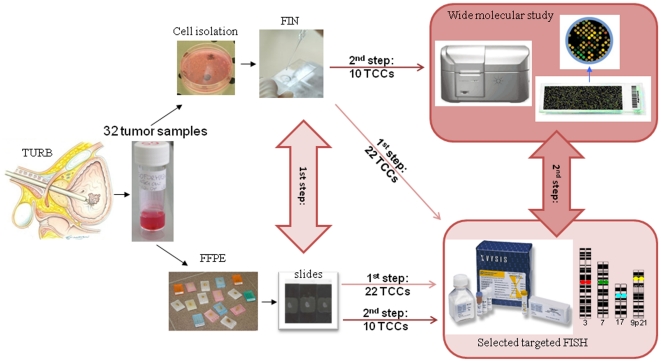
Methodological approach of this study. The two step strategy of analysis applied in this study.

The first goal of this study was to evaluate the performance of a targeted test in two different types of material derived from the same tumor. In the first step we compared the results of UroVysion test performed on Freshly Isolated interphasic Nuclei (FIN) and on FFPE tissues from 22 TCCs (Figure 1). Furthermore, a second goal was to assess the concordance between array-CGH profiles and the targeted chromosomal profiles, in order to evaluate whether UroVysion is an adequately sensitive method for the identification of selected aneuploidies and nonrandom CNAs in TCCs. The second step of comparison was applied on an additional set of 10 TCCs, between data derived from either array-CGH on FIN and from UroVysion analysis on FFPE tissues (Figure 1).

## Results

### First Step of analysis: comparison between UroVysion data from FIN and FFPE

TCC can be distinguished in high or low grade (HG or LG) and in muscle invasive or not (IN or NI). In the first step of analysis 22 TCCs (9 LGNI, 1 LGIN, 3 HGNI, 9 HGIN) were analyzed by UroVysion test applied in duplicate from the same biopsy on FFPE and FIN samples (Figure 1).

In the analysis based on the multinomial model, FIN data were generally comparable to those extracted from FFPE counterpart in LGNI group, in terms of percentages of loss, disomy and gain (Table 1; for a detailed list see Table S2). By contrast, in HGNI group the two types of analysis generated concordant results only for CEP 3 (Chromosome Enumeration Probe 3). Indeed in CEP 7 and CEP17, FIN tended to detect a lower percentage of both loss (1,7 vs 10 for CEP 7; 6 vs 11,3 for CEP17) and disomy (42,7 vs 62,3 for CEP7; 48,3 vs 60,7 for CEP17); consequently a higher percentage of gain was reported (55,7 vs 27,7 for CEP7; 45,7 vs 28 for CEP 17). Finally, for Locus Specific Identifier (LSI) 9p21 FIN tended to detect a higher percentage of both disomy (58,3 vs 20,0) and gain (10,3 vs 8,7) but a lower percentage of loss (31,3 vs 71,3). On the other hand, in HGIN group the two types of analysis generated discordant results only for CEP 3: FIN tended to detect a higher percentage of loss (14,9 vs 2,2) and a lower percentage of gain (48,1 vs 62,5) than FFPE.

**Table 1:** Percentages of loss, disomy, gain by type of tumor, probe analyzed and test applied.

LG NI								
Probe	Test	% loss	(95% CI)	% diso	(95% CI)	% gain	(95% CI)	p-value
**CEP 3**	FIN	13,2	(6,7 ; 24,3)	57,9	(32,2 ; 79,9)	28,9	(23,5 ; 35,0)	0,520
	FFPE	10,0	(4,2 ; 22,1)	55,0	(35,2 ; 73,3)	35,0	(30,5 ; 39,7)	
**CEP 7**	FIN	28,0	(14,6 ; 47,0)	57,8	(35,6 ; 77,2)	14,2	(13,1 ; 15,4)	0,134
	FFPE	10,4	(5,0 ; 20,6)	70,2	(57,2 ; 80,6)	19,3	(17,6 ; 21,1)	
**CEP 17**	FIN	14,4	(9,2 ; 21,7)	71,3	(56,7 ; 82,5)	14,4	(12,7 ; 16,1)	0,133
	FFPE	9,1	(5,1 ; 15,7)	68,9	(50,9 ; 82,5)	22,0	(18,9 ; 25,5)	
**LSI 9p21**	FIN	84,9	(53,6 ; 96,5)	14,5	(3,2 ; 46,6)	0,6	(0,6 ; 0,6)	0,323
	FFPE	73,2	(55,1 ; 85,9)	24,0	(10,9 ; 45,0)	2,8	(2,7 ; 2,8)	

HG NI								
Probe	Test	% loss	(95% CI)	% diso	(95% CI)	% gain	(95% CI)	p-value
**CEP 3**	FIN	5,3	(1,2 ; 20,7)	48,0	(16,6 ; 81,0)	46,7	(32,9 ; 61,0)	0,307
	FFPE	11,7	(5,5 ; 23,0)	56,3	(33,3 ; 77,0)	32,0	(27,3 ; 37,1)	
**CEP 7**	FIN	1,7	(0,5 ; 5,2)	42,7	(16,5 ; 73,8)	55,7	(38,6 ; 71,5)	<0.001*
	FFPE	10,0	(4,9 ; 19,4)	62,3	(31,6 ; 85,6)	27,7	(21,7 ; 34,6)	
**CEP 17**	FIN	6,0	(3,0 ; 11,5)	48,3	(13,7 ; 84,7)	45,7	(28,3 ; 64,2)	0,020*
	FFPE	11,3	(5,8 ; 21,1)	60,7	(24,3 ; 88,1)	28,0	(20,3 ; 37,3)	
**LSI 9p21**	FIN	31,3	(18,0 ; 48,7)	58,3	(30,1 ; 82,0)	10,3	(8,7 ; 12,2)	<0.001*
	FFPE	71,3	(56,3 ; 82,8)	20,0	(6,8 ; 46,2)	8,7	(8,0 ; 9,4)	

HG IN								
Probe	Test	% loss	(95% CI)	% diso	(95% CI)	% gain	(95% CI)	p-value
**CEP 3**	FIN	14,9	(7,2 ; 28,4)	36,9	(22,0 ; 54,9)	48,1	(44,2 ; 52,1)	0,038*
	FFPE	2,2	(0,7 ; 6,9)	35,3	(24,0 ; 48,5)	62,5	(55,0 ; 9,4)	
**CEP 7**	FIN	8,9	(5,0 ; 15,6)	50,9	(29,6 ; 72,0)	40,1	(32,7 ; 48,1)	0,741
	FFPE	3,6	(1,5 ; 8,5)	54,0	(36,7 ; 70,4)	42,4	(35,7 ; 49,3)	
**CEP 17**	FIN	18,5	(9,0 ; 34,3)	49,9	(23,4 ; 76,4)	31,6	(25,4 ; 38,5)	0,244
	FFPE	6,9	(2,7 ; 16,8)	47,5	(27,0 ; 69,0)	45,5	(37,6 ; 53,7)	
**LSI 9p21**	FIN	63,3	(40,2 ; 81,6)	20,9	(2,5 ; 73,3)	15,7	(13,1 ; 18,8)	0,934
	FFPE	64,5	(39,2 ; 83,6)	20,0	(2,4 ; 71,5)	15,5	(13,5 ; 17,8)	

p-values refer to the comparison between the two type of analysis obtained by a multinomial model with 95% confidence interval.

**Figure 2:**
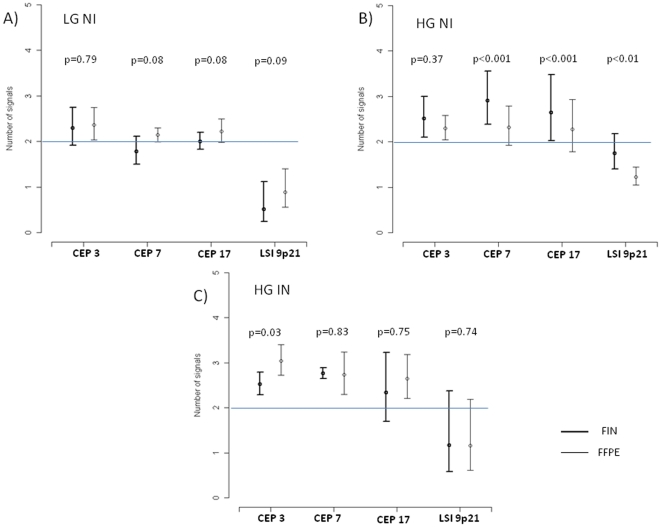
Statistical analysis by a Poisson model. Estimated count of signals observed in each probe (with 95% confidence interval) obtained in each type of tumor, accounting for clustering. The reported p-values refer to the comparison between FFPE and FIN methods. Panel A =  LGNI (9 pts); panel B =  HGNI (3 pts); panel C =  HGIN (9 pts).

These results were confirmed in the more refined analysis by a Poisson model (Figure 2). In LGNI group, the average number of signals for CEP3, CEP7, CEP17 and for the 9p21 region was 2.3, 1.8, 2.0, 0.5 (in FIN samples) and 2.4, 2.1, 2.2, 0.9 (in FFPE) with no significant difference between the two types of test (Figure 2, A). On the other hand, in HGNI group the average number of signals for CEP3, CEP7, CEP17 and for the 9p21 region was 2.5, 2.9, 2.7, 1.8 (in FIN samples) and 2.3, 2.3, 2.3, 1.2 (in FFPE) with statistically significant differences between the two tests except for CEP3 (Figure 2, B). Conversely, in HGIN group, the average number of signals for CEP3, CEP7, CEP17 and for the 9p21 region was 2.5, 2.7, 2.3, 1.2 (in FIN) and 3.0, 2.7, 2.7, 1.2 (in FFPE samples), with significant difference for CEP3 (Figure 2, C).

### Genomic copy number alterations (CNAs) in freshly isolated nuclei (FIN) by array-CGH

**Figure 3:**
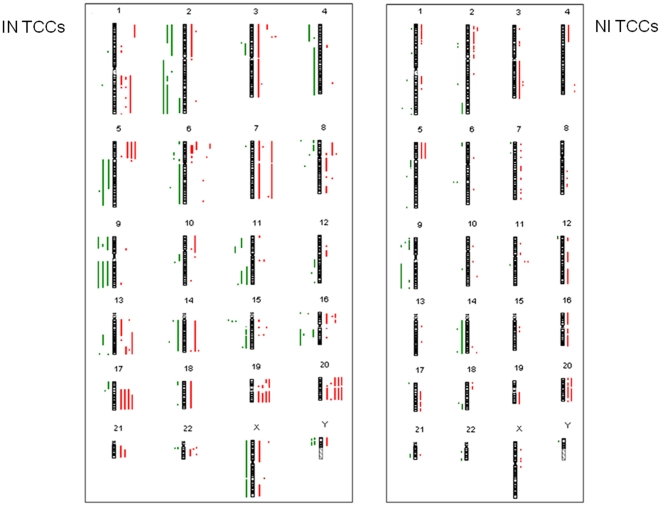
CNA collection evidenced by array-CGH. CNAs of 10 TCCs samples: 6 infiltrating tumors (IN-TCCs: 70CR09, 81CR09, 04CR10, 09CR10, 10CR10, 26CR10) on the left, and 4 non- infiltrating tumors (NI-TCCs: 28CR09, 75CR09, 80CR09, 82CR09) on the right. Each dot/bar corresponds to one sample. Losses are evidenced in green while gains in red.

In the second step of our analysis we first performed array-CGH on an additional set of 10 TCCs (6 HGIN, 1 HGNI, 3 LGNI), in order to detect CNAs between tumor and reference DNA. The most frequent CNAs are summarized in Table 2 (detailed form in Table S3). We classified the samples into two categories: infiltrating tumors (IN-TCCs: 70CR09, 81CR09, 04CR10, 09CR10, 10CR10, 26CR10) and non infiltrating tumors (NI-TCCs: 28CR09, 75CR09, 80CR09, 82CR09) (Figure 3). In general, as expected, IN-TCCs have many more CNAs than NI-TCCs. 20q gain was shared by 4/6 IN-TCC tumors, while 2/4 NI-TCCs; 3p25.2 and 17q21 gains by 4/6 IN-TCC tumors and 1/4 NI-TCCs; 5p and 20p gain by 3/6 IN-TCC and 2/4 NI-TCCs; 6p22.3 and 11q13 was shared only by IN-TCC (3/6 and 2/6 respectively); finally 3q and 8q were in 1/6 IN-TCCs and 1/4 NI-TCCs. For the losses: 9p and 9p21 were in 4/6 and 3/6 IN-TCCs while in 2/4 and 3/4 NI-TCCs; 9q32-q34 were in 3/6 IN-TCCs and 2/4 of NI-TCCs; 2q loss were in 3/6 IN-TCCs and 1/4 NI-TCCs; 8p loss only in 2/6 IN-TCCs.

**Table 2:** The most frequent CNAs evidenced by array-CGH in this study and comparison with data from literature.

		LGNI			HGNI	HGIN					
GAIN	ref	75CR09	80CR09	82CR09	28CR09	04CR10	09CR10	10CR10	26CR10	70CR09	81CR09
3p25	18				+		+	+		+	+
3q	18,19				+		+				
5p	18,19		+		+			+		+	+
6p22.3	18,19					+	+				+
8q	18,19				+		+				
11q13	18						+	+			
17q21	18				+	+	+	+		+	
20p	18,19		+		+			+	+	+	
20q	18,19		+		+		+	+	+	+	
**LOSS**	**ref**	**75CR09**	**80CR09**	**82CR09**	**28CR09**	**04CR10**	**09CR10**	**10CR10**	**26CR10**	**70CR09**	**81CR09**
2q	18,19				+		+		+		+
8p	18,19						+	+			
9p	19	+			+		+	+	+		+
9p21	18	+	+		+		+	+	+		
9q32-q34	19		+		+		+	+	+		

To identify possible enrichment of functional groups in the genes within regions with gain and loss of HGIN and LGNI tumors, a gene ontology annotation analysis was performed using the GOstat software. For HGIN emerged a statistically significant under-representation (p<0.05) of genes involved in cell differentiation, in cell cycle and in positive regulation of apoptosis and programmed cell death; in addition a statistically significant over-representation of genes involved in cell proliferation and in regulation of apoptosis. On the other hand the analysis evidenced for LGNI tumors a statistically significant under-representation of genes involved in induction of apoptosis and programmed cell death (Table S4).

### Second Step of analysis: comparison between array-CGH profiles on FIN and UroVysion data on FFPE

**Figure 4:**
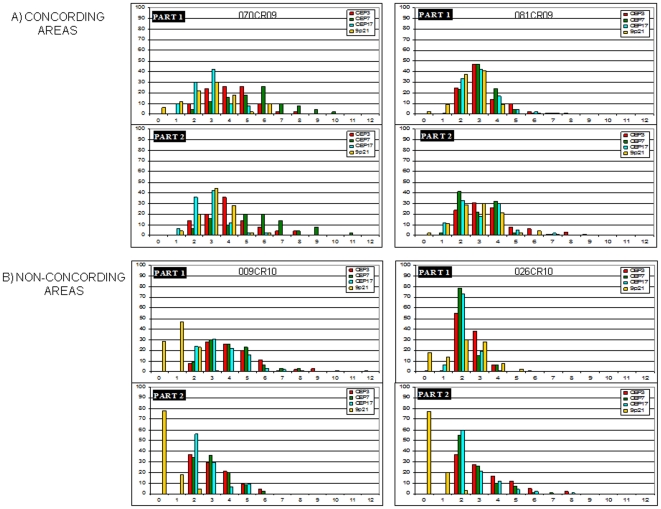
Examples of concordant and non-concordant results of Urovysion test. Comparison between results on two selected tumoral areas of the same section of FFPE: (A): the two most concordant tumors (070CR09 and 081CR09); (B): the two most discordant tumors (009CR10 and 026CR10).

We next performed FISH analysis by means of Urovysion test on the additional set of 10 TCCs analyzed by array-CGH; when possible, two tumoral areas of the same section were scored in order to increase the number of cell analyzed and to have data as representative as possible, given the well-known heterogeneity in this type of cancer. For each probe a statistical analysis was performed to verify that the signal counts on 100 cells were different considering the two areas separately or mixing them together. Concordant results between the two tumoral areas were reported in two HGIN cases (070CR09 and 081CR09) (Figure 4, A); conversely, statistically significant contrasting results (p< 0.05) were reported in two HGIN cases (009CR10 and 026CR10) (Figure 4, B); in the remaining six cases statistically significant differences between the two tumoral areas were evidenced for one (010CR10), two (028CR09 and 080CR09) or three probes (004CR10) (see also Table S5). These data stressed the overall high intra-tumor heterogeneity of these samples.

**Figure 5:**
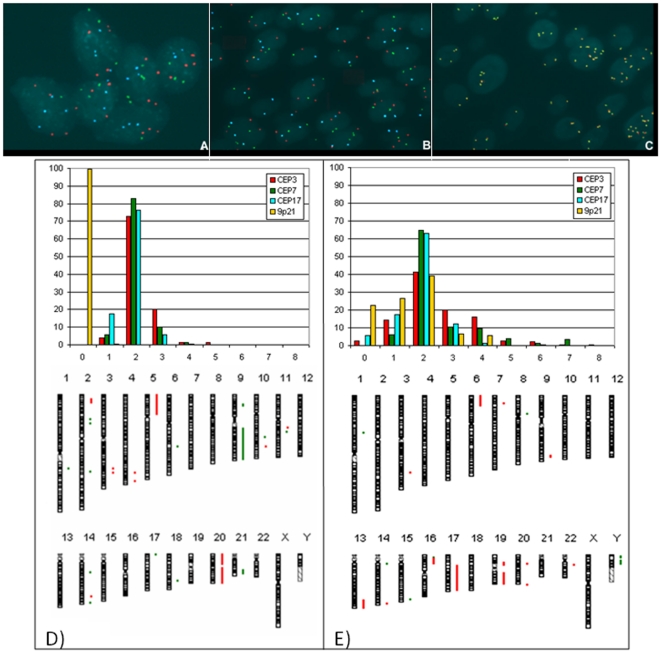
Examples of FISH analysis. Urovysion test applied to: (A): FIN sample 032CR07 (HG NI); (B): FFPE sample 080CR09 (LG NI). (C) FISH with *PPAR*γ probe on 028CR09 (HGNI). Urovysion versus array-CGH data: example of concordant data (D), (sample 080CR09); and non-concordant data (E), (sample 004CR10).

Then, we made an attempt to compare the UroVysion data on FFPE just reported with the array-CGH profiles from the 10 TCCs, described above. For this purpose for each sample we extrapolated the results for array-CGH analysis corresponding to the four UroVysion targeted chromosomes and compared them with FISH data (Table 3). Full concordance was found only for 28CR09 (HGNI) and 09CR10 (HGIN) (gray areas in Table 3). However, for the other tumors, a fairly good correlation has been observed between the two techniques; ie for tumors 010CR10 and 070CR09 (both HGIN) the concordance was evidenced for 3/4 targeted chromosomes. See Fig 5 for two examples of more concordant (D) and less concordant (E) data. The greater concordance was seen for chromosome 3 (7/10), while the other targeted chromosomes showed a reasonable correlation (6/10). For example, in 082CR09 (LGNI) and in 04CR10 (HGIN), 9p21 losses were evidenced only by FISH analysis; on the other hand the amplification at locus 3p25 for 028CR09 (HGNI) and for 070CR09 (HGIN) emerged only from array-CGH data. In order to validate array-CGH data and to distinguish a polysomy of chromosome 3 from a true amplification, FISH analysis was performed with both Urovysion test assay and the dual-color split probe PPARγ (3p25), on two consecutive FFPE sections of 028CR09 (Figure 5, C). A statistical analysis of signal counts on 100 nuclei assessed the true amplification at 3p25 respect to a polysomy of chromosome 3 (*t test*: p<0.01).

**Table 3:** Comparison between results of Urovysion FISH (in two selected tumoral areas of the same FFPE section) and array-CGH.

SAMPLE		UROVYSION AREA 1		UROVYSION AREA 2		Array-CGH	
		LOSS%	GAIN%	LOSS%	GAIN%	LOSS%	GAIN%
075CR09 LG NI	CR3*	12	29	na	na	-	-
	CR7**	2	24	na	na	-	-
	CR17*	25	12	na	na	-	-
	9p21**	46	5	na	na	mosaic 48%	-
080CR09 LG NI	CR3**	4	27	4	16	-	3q25.2, 1.2 Mb, amplification 3q26.1, 1.8 Mb, non mosaic
	CR7	7	8	4	12	-	-
	CR17**	25	3	10	8	17p13.3, 0.07 Mb, mosaic 87%	-
	9p21	100	0	100	0	non mosaic	-
082CR09 LG NI	CR3*	1	29	na	na	-	-
	CR7**	0	14	na	na	-	-
	CR17**	7	7	na	na	-	-
	9p21*	93	1	na	na	-	-
028CR09 HG NI	CR3	1	56	0	53	-	3p25.2-p25.1, 2 Mb, amplification 3p21.31, 3 Mb, mosaic 62% 3q11.2-q29, 99.5 Mb, mosaic 51%
	CR7	3	57	0	33	7p22.3, 0.1 Mb, non mosaic	7p22.2-p22.1, 2 Mb, mosaic 80% 7p15.3, 0.3 Mb, mosaic 62% 7p14.2, 0.28 Mb, non mosaic 7q11.21-q11.23, 1.2 Mb, mos 77% 7q21.3-q22.2, 7.6 Mb, mosaic 62% 7q32.1-q32.2, 1 Mb, mosaic 85% 7q33.34, 2.9 Mb, mosaic 77% 7q36.1, 0.7 Mb, non mosaic
	CR17	0	53	1	39	-	17q11.1-q25.3, 56 Mb, mosaic 85%
	9p21	55	4	78	1	non mosaic	-
004CR10 HG IN	CR3**	20	18	14	28	-	3q25.32-q26.1, 2.2 Mb, mos 51%
	CR7*	8	12	4	24	-	7p21.1, 3.6 Mb, mos 68%
	CR17*	28	4	18	21	-	17p11.2, 0.4 Mb, mosaic 48% 17q11.2, 2.9 Mb, mosaic 45% 17q12-q25.3, 49 Mb, mosaic 39%
	9p21**	70	0	28	13	-	-
009CR10 HG IN	CR3	0	52	0	38	-	3p26.3-p11.1, 90 Mb, mosaic 75% 3q11.2-q29, 104.2 Mb, mosaic 60%
	CR7	0	56	0	44	-	7p22.3-p11.2, 56.2 Mb, mosaic 39% 7q11.21-q36.3, 96.3 Mb, mosaic 66%
	CR17	0	49	0	38	17p13.3-p11.2, 21.8 Mb, mosaic 33%	17q11.1-q25.3, 55.8 Mb, mosaic 28%
	9p21	76	1	96	0	mosaic 33%	-
010CR10 HG IN	CR3	3	31	2	49	3p21.31-p12.1, 35.1 Mb, mosaic 28%	3p16.3-p24.3, 6.6 Mb, mosaic 68%
	CR7**	0	33	1	31	7p21.1, 0.3 Mb, mosaic 91%	-
	CR17	10	25	9	25	-	17q11.1-q25.3, 55.7 Mb, mosaic 30%
	9p21	91	1	99	0	non mosaic	-
026CR10 HG IN	CR3**	0	38	0	39	3p21.31-p21.1, 4.2 Mb, mosaic 27%	-
	CR7*	1	15	0	34	-	-
	CR17*	7	19	0	25	-	-
	9p21	32	30	97	0	mosaic 68%	-
070CR09 HG IN	CR3	0	52	0	38	-	3p25.2-p25.1, 0.7 Mb, amplification
	CR7	0	44	0	58	-	7p22.3-q36.3, 158 Mb, mosaic 66%
	CR17	10	50	6	44	17q11.2-q12, 1 Mb, mosaic 48%	17q21.31-q25.3, 40 Mb, mosaic 46%
	9p21**	18	32	4	46	-	-
081CR09 HG IN	CR3	0	58	0	41	-	3p25.2, 0.06 Mb, mosaic 62%
	CR7**	0	52	2	25	-	-
	CR17**	1	47	12	25	-	-
	9p21**	11	42	13	33	-	-

LOSS: number of signals: 0 and 1; DISOMY: number of signals even (2, 4, 6, 8, 10, 12); GAIN number of signals odd (3, 5, 7, 9, 11).

Gray: CNA detected by both techniques and by both tumoral FISH areas.

**: CNA detected by both techniques but by only one tumoral FISH area; or by both tumoral FISH area but not by array-CGH.

*: CNA detected by only array-CGH, or by only one tumoral FISH area.

Positive FISH if at least 25% of cells detected the CNA.

Na: not available.

## Discussion

Despite the extensive research into genetic alterations of bladder cancer and detailed models which link such changes to tumor initiation and progression [20]–[22], there are few reliable markers to distinguish tumors with aggressive characteristics at the time of early diagnosis and we are still looking for the method of election to detect them. In this regard, a recent prospective study has even suggested that cystoscopy alone remains the most cost-effective strategy for detecting recurrence of bladder cancer not invading the muscle [28]. However, in contrast to what previously reported by others [29], several authors claimed the same conclusion [30], and the role of Urovysion in suspicious urine specimens remained questionable, especially in view of its high cost.

The development of array-CGH led to the possibility to analyze the whole genome in a single experiment, suggesting its possible application in screening/surveillance programs of cancer patients. In the case of bladder cancer, array-CGH would give the possibility to analyze the DNA from a biopsy of the tumor, while by Urovysion urine specimens are usually analyzed.

The main drawbacks of this technique are that, even if it is specific and sensitive, it is invasive and still expensive. Furthermore, to date there are no sufficient data to support the use of array-CGH in this kind of programs, but it could be interesting to apply this technique for patients' categories with high cancer risk.

The multitarget Urovysion assay has been developed for the detection of TCC in urine specimens [7]. The optimal FISH probe set was determined by testing different probes for TCC detection in urine from patients with bladder cancer and selecting those that were either the most sensitive individually or that complemented other probes to enhance the overall sensitivity of the test. The CEP probes and LSI 9p21 were complementary because the CEP probes detect hyperdiploidy, common in carcinoma *in situ* and invasive TCC, while the LSI 9p21 probe detects deletions of the 9p21 band, common in non-invasive TCC [7]. It has been previously suggested that a false-negative FISH result represents mostly low-grade TCC that do not shed tumor cells into the urine or do not exhibit the chromosomal alterations that are detected by the assay [11]. Another limit, and another possible explanation for false-negative FISH results, might be attributed to the low number of neoplastic cells present in the specimens [30].

In the first step of this study we compared the performance of this multitarget assay for the detection of bladder tumor cells both in FIN, without histological diagnosis and even with a low number of neoplastic cells, and in FFPE tissue. Our analysis evidenced a good correspondence of Urovysion FISH data between FIN and FFPE for LGNI and HGIN tumors; in particular, in the former group, FIN tended to detect a smaller number of signal respect to FFPE, while in the latter group an opposite tendency was appreciated. For HGNI TCCs, significant differences emerged for three targeted probes, but it could be due to the low number of samples of this group. The performance of this targeted test is therefore sufficiently acceptable also on FIN samples; furthermore the same CNAs were faithfully reflected by the analysis on FFPE. It remains to investigate whether it is an efficient method to detect the most representative and effective CNAs of TCCs. For this purpose, in the second step of this study, array-CGH was performed on 10 additional TCCs to dissect the spectrum of alterations in bladder cancer and to identify recurrent aberrations that may contain cancer-related genes.

We detected numerous genetic changes by array-CGH: the most frequent loss involved chromosome 9p-arm while the most frequent gain involved chromosome 20q-arm, as previously reported by others [5], [6], [14], [18], [19]. Surprisingly, we didn't find a high percentage of tumors with gain of 6p22.3 and 8q reported in other studies [14], [18], [19]. LOH and under-representation of chromosome 9 is the most frequently described genetic alteration in TCC (>50%). The common loss of an entire copy of chromosome 9 indicates the presence of tumor suppressor genes both on 9p and 9q, and candidate genes have been identified in several regions including 9p21 (*CDKN2A*), 9q12-13 (*PTCH*), 9q32-33 (*DBC1*) and 9q34 (*TSC1*). In this study, we observed complete or partial loss of 9p and/or 9q in 7/10 tumors, in both HG and LG. Moreover, in some HG we observed a gain for this locus, even if this could be due to chromosome 9 polyploidy (as the sign of chromosomal instability). The most frequent gain is 20q (6 tumors), in accordance with data previously reported in many other cancers, including bladder, colon, ovarian and breast [31]. Association of 5p and 20q gains, found in 3 HG tumors, reported by Bruch [32], could be associated with progression. Finally, gain of 17q21 is identified only in HG tumors, suggesting a possible role in tumor progression.

The most interesting point of this study is the comparison of array-CGH data and Urovysion FISH data. Indeed, we evidenced not only a high intra and inter-tumor heterogeneity in FFPE material, as emerged from the analysis of two different tumoral areas of the same tumor; we also found some discrepancies in the two techniques that could be partially ascribed to a possible masking effect from normal cells or to a compensatory effect derived from the great tumor heterogeneity. This heterogeneity has already been described by our group in bladder cancer stem-like cells that are genetically different [33]. We can suggest that this diversity generates viable and clonally related subpopulations that become heterogeneous in the same tumor.

The overall array-CGH data stressed once again on the presence of frequent alterations (i.e. 20p and 5p gains) that cannot be detected by Urovysion assay. A further advantage of using an integrated technical approach emerged for 028CR09 sample: the amplification of 3p evidenced by array-CGH was studied by FISH with Urovysion assay and a LSI 3p probe. Through the integration of multiple methods, we were able to discriminate the true amplification from a chromosome 3 polysomy. This locus includes the peroxisome proliferator-activated receptor gamma (*PPARG*), a ligand activated transcription factor implicated in the regulation of proliferation and differentiation of urothelium [34], [35].

In conclusion, considerable effort is still required to define the genes underlying the chromosomal abnormalities to a better understand of the genetic mechanisms in order to develop new therapeutic strategies. Our results confirmed the importance of global genomic screening methods, that is array based CGH, to comprehensively determine the genomic profiles of large series of TCCs tumors. However, this technique has yet some limitations, such as not being able to detect low level mosaicism, or not detecting any change in the number of copies for a kind of compensatory effect due to the presence of high cellular heterogeneity. Thus, it is still advisable to use complementary techniques such array-CGH and FISH, as the former is able to detect alterations at the genome level not excluding any chromosome, but the latter is able to maintain the individual data at the level of single cells, even if it focuses on few genomic regions.

## Materials and Methods

A detailed form can be found in Materials and Methods S1.

This study was approved and founded by Direzione Generale Sanità Regione Lombardia and presented by General Director and ethic commitment of ICP Hospital Bassini. Written informed consent was obtained from the study participants before tissue collection.

### Patients and samples

A total of 32 tumor samples (28 men and 4 women) were obtained by transurethral resection in a consecutive series of patients newly diagnosed with TCCs at a single center (Table S1). Informed consent was obtained before tissue collection. Staging and grading were done according to the World Health Organization Consensus Classification [1]. They were distinguished in high or low grade (HG or LG) and in muscle invasive or not (IN or NI).

### Fluorescence in situ hybridization

For FIN, biopsies were cut up and cultured in RPMI-1640 (Euroclone Spa) supplemented with 20% FCS for 24 hours. Pieces were subjected to hypotonic treatment and fixed with 3:1 methanol:acetic acid. Single cells isolated from biopsies with acetic acid 60%, were spotted on slides and let dry. For FFPE, tissue were fixed according to standard procedures.

Pretreatment and FISH analysis were performed on both nuclei isolated from FIN and FFPE samples using UroVysion bladder cancer kit (Vysis, Wiesbaden, Germany), according to the manufacturer's instructions.

After hybridization the unbound probes were removed by a series of washes and the nuclei were counterstained with 4′,6-diamidino-2-phenylindole (DAPI).

At least 100 cells for each preparation were scored and the signals were divided according to loss (number of signals/cell <2), disomy (number of signals/cell  = 2) and gain (number of signals/cell >2).

For locus 3p25 FISH analysis on FFPE was performed using Poseidon™ Repeat Free™ PPARγ (3p25) Break probe (Kreatech Diagnostics, Amsterdam, Netherlands). The statistical significance of differences between chromosome 3 polisomy and 3p25 amplification was evaluated by Student's t test on separate counts of 100 nuclei. Differences were considered as statistically significant with p<0.01.

All digital images were captured using a Leitz microscope (Leica DM 5000B) equipped with a charge coupled device (CCD) camera and analyzed by means of Chromowin software (Tesi Imaging, Milan, Italy).

### Array-CGH

For array-CGH analysis, genomic DNA was extracted from fresh biopsies after enzymatic digestion with collagenase H (Roche, Mannheim, Germany) and proteinase K (Roche, Mannheim, Germany) and purified using phenol/chloroform (Carlo Erba, Milan, Italy). Sample preparation, slide hybridization, and analysis were performed using SurePrint G3 Human CGH Microarray 8x60K (Agilent, Santa Clara, CA) according to the manufacturer's instructions. Sex-matched commercial DNA samples (Promega) were used as reference DNA during array-CGH. The arrays were scanned at 2-µm resolution using Agilent microarray scanner and analyzed using Feature Extraction v10.7 and Agilent Genomic Workbench v5.0 softwares. The Aberration Detection Method 2 (ADM2) algorithm prompted by Genomic Workbench software was used to compute and assist the identification of aberrations for a given sample (threshold = 5; log2 ratio = 0.3). To calculate the estimated percentage of mosaicism we used the formula determined by Cheung SW et al. [36].

### Gene ontology analysis

To analyze which ontology classes were over- and under- represented among the genes delineated within gain and loss regions detected by array-CGH, the GOstat software (available at http://gostat.wehi.edu.au/) was used [37] based on AmiGO (the Gene Ontology database) version 1.8.

### Statistical analysis

Cases were described by calculating the proportions of loss, disomy and gain on the total of at least 100 cells, specifically for type of analysis (FFPE and FIN), and probe of UroVysion test.

A multinomial model accounting for the presence of clustering was used to estimate for each type of tumor and type of analysis, the overall proportion of loss, disomy and gain with 95% confidence intervals. This model was also used to compare the overall proportions of loss, disomy and gain detected by the two types of analysis.

A Poisson model based on logarithmic transformation of counts in the presence of clustering was used to estimate the number of signals detected by each type of analysis with 95% confidence intervals. This model enabled also to compare the number of signals across the two types of analysis (FFPE and FIN).

## Supporting Information

Table S1 **Clinicopathologic characteristics of 32 tumor samples of the study.** Histology/Grade and phase of study are indicated.
(DOC)

Table S2 **UroVysion test results on freshly isolated interphasic nuclei (FIN) and on formalin fixed paraffin embedded nuclei (FFPE).**
(DOC)

Table S3 **Copy number alterations (CNA) shared (plus sign) among 10 TCC samples analyzed by array-CGH.** NI-TCCs are indicated in italics; IN-TCCs are indicated in bold. For Histology/Grade see Table S1.
(DOC)

Table S4 **Gene Ontology.** I. Statistically significant (p<0.05) under-representation of gene ontology (GO) categories in HG IN tumors. II. Statistically significant (p<0.05) over-representation of gene ontology (GO) categories in HG IN tumors. III. Statistically significant (p<0.05) under-representation of gene ontology (GO) categories in LG NI tumors.
(DOC)

Table S5 **Urovysion data.** I. Comparison between Urovysion data in two different tumoral areas of the same section II. Comparison between Urovysion data in two different tumoral areas of the same section
(DOC)

Materials and Methods S1 (DOC)
