# Supplementary material for: Chromosomal Aberrations in Bladder Cancer: Fresh versus Formalin Fixed Paraffin Embedded Tissue and Targeted FISH versus Wide Microarray-Based CGH Analysis
Source: PLoS One. 2011 Sep 1;6(9):e24237. doi: 10.1371/journal.pone.0024237 (PMC3164716; doi:10.1371/journal.pone.0024237)
Supplement: Table S1 — Clinicopathologic characteristics of 32 tumor samples of the study. Histology/Grade and phase of study are indicated. (DOC) [file pone.0024237.s001.doc]

| Table S1. Clinicopathologic characteristics of 32 tumor samples of the study. Histology/Grade and phase of study are indicated. | | | | |
| --- | --- | --- | --- | --- |
| **Case** | **Age** | **Sex** | **Histology/Grade** | **Phase of Study** |
| 006CR07 | 77 | M | TCC LG NI | First step (22 TCCs) |
| 007CR07 | 79 | M |
| 020CR07 | 65 | M |
| 021CR07 | 78 | M |
| 024CR07 | 70 | M |
| 030CR07 | 70 | M |
| 039CR07 | 75 | M |
| 048CR07 | 75 | M |
| 050CR07 | 70 | M |
| 051CR07 | 73 | M | TCC LG IN |
| 016CR06 | 70 | M | TCC HG NI |
| 032CR07 | 65 | M |
| 034CR07 | 78 | F |
| 010CR06 | 54 | F | TCC HG IN |
| 013CR06 | 81 | M |
| 014CR06 | 54 | M |
| 017CR06 | 67 | M |
| 019CR06 | 71 | M |
| 037CR07 | 85 | M |
| 038CR07 | 65 | M |
| 040CR07 | 88 | M |
| 045CR07 | 78 | M |
| 75CR09 | 81 | M | TCC LG NI | Second step (10 TCCs) |
| 80CR09 | 78 | M |
| 82CR09 | 56 | F |
| 28CR09 | 67 | M | TCC HG NI |
| 04CR10 | 76 | M | TCC HG IN |
| 09CR10 | 82 | F |
| 10CR10 | 93 | M |
| 26CR10 | 85 | M |
| 70CR09 | 73 | M |
| 81CR09 | 67 | M |
