# Supplementary material for: Chromosomal Aberrations in Bladder Cancer: Fresh versus Formalin Fixed Paraffin Embedded Tissue and Targeted FISH versus Wide Microarray-Based CGH Analysis
Source: PLoS One. 2011 Sep 1;6(9):e24237. doi: 10.1371/journal.pone.0024237 (PMC3164716; doi:10.1371/journal.pone.0024237)
Supplement: Table S2 — UroVysion test results on freshly isolated interphasic nuclei (FIN) and on formalin fixed paraffin embedded nuclei (FFPE). (DOC) [file pone.0024237.s002.doc]

| Table S2. UroVysion test results on freshly isolated interphasic nuclei (FIN) and on formalin fixed paraffin embedded nuclei (FFPE). | | | | | | | | | | | | | | |
| --- | --- | --- | --- | --- | --- | --- | --- | --- | --- | --- | --- | --- | --- | --- |
| **Histology/Grade** | | | **CEP 3** | | | **CEP 7** | | | **CEP 17** | | | **LSI 9p21** | | |
|  | Case | Test | % Loss | % Disomy | % Gain | % Loss | % Disomy | % Gain | % Loss | % Disomy | % Gain | % Loss | % Disomy | % Gain |
| **TCC LG NI** | 006CR07 | FFPE | 0 | 72 | 28 | 0 | 87 | 13 | 1 | 90 | 9 | 84 | 16 | 0 |
|  |  | FIN | 8 | 72 | 20 | 50 | 50 | 0 | 24 | 76 | 0 | 96 | 4 | 0 |
|  | 007CR07 | FFPE | 41 | 59 | 0 | 5 | 89 | 6 | 6 | 92 | 2 | 100 | 0 | 0 |
|  |  | FIN | 40 | 55 | 5 | 3 | 83 | 14 | 12 | 84 | 4 | 99 | 0 | 1 |
|  | 020CR07 | FFPE | 2 | 52 | 46 | 1 | 71 | 28 | 6 | 70 | 24 | 61 | 35 | 4 |
|  |  | FIN | 1 | 82 | 17 | 54 | 46 | 0 | 3 | 91 | 6 | 99 | 1 | 0 |
|  | 021CR07 | FFPE | 0 | 74 | 26 | 0 | 89 | 11 | 1 | 88 | 11 | 100 | 0 | 0 |
|  |  | FIN | 24 | 67 | 9 | 68 | 30 | 2 | 28 | 65 | 17 | 100 | 0 | 0 |
|  | 024CR07 | FFPE | 7 | 48 | 45 | 10 | 72 | 18 | 8 | 77 | 15 | 50 | 46 | 4 |
|  |  | FIN | 5 | 61 | 34 | 49 | 41 | 10 | 18 | 65 | 17 | 79 | 17 | 4 |
|  | 030CR07 | FFPE | 22 | 66 | 12 | 35 | 61 | 4 | 28 | 69 | 3 | 58 | 39 | 3 |
|  |  | FIN | 8 | 81 | 11 | 0 | 77 | 23 | 9 | 89 | 2 | 4 | 96 | 0 |
|  | 039CR07 | FFPE | 4 | 24 | 72 | 26 | 32 | 42 | 10 | 14 | 76 | 31 | 60 | 9 |
|  |  | FIN | 23 | 76 | 1 | 0 | 93 | 7 | 4 | 93 | 3 | 98 | 2 | 0 |
|  | 048CR07 | FFPE | 2 | 50 | 48 | 4 | 54 | 42 | 7 | 47 | 46 | 99 | 1 | 0 |
|  |  | FIN | 5 | 19 | 76 | 20 | 53 | 27 | 8 | 34 | 58 | 99 | 1 | 0 |
|  | 050CR07 | FFPE | 11 | 51 | 38 | 14 | 76 | 10 | 15 | 73 | 12 | 82 | 13 | 5 |
|  |  | FIN | 2 | 14 | 84 | 19 | 43 | 38 | 28 | 47 | 25 | 95 | 5 | 0 |
| **TCC LG IN** | 051CR07 | FFPE | 28 | 50 | 22 | 7 | 52 | 41 | 19 | 42 | 39 | 96 | 4 | 0 |
|  |  | FIN | 0 | 2 | 98 | 9 | 22 | 69 | 3 | 20 | 77 | 98 | 2 | 0 |
| **TCC HG NI** | 016CR06 | FFPE | 8 | 59 | 33 | 11 | 76 | 13 | 19 | 72 | 9 | 65 | 17 | 18 |
|  |  | FIN | 15 | 73 | 12 | 4 | 71 | 25 | 11 | 87 | 2 | 20 | 49 | 31 |
|  | 032CR07 | FFPE | 22 | 63 | 15 | 17 | 70 | 13 | 12 | 77 | 11 | 88 | 11 | 1 |
|  |  | FIN | 1 | 50 | 49 | 1 | 48 | 51 | 3 | 53 | 44 | 51 | 49 | 0 |
|  | 034CR07 | FFPE | 5 | 47 | 48 | 2 | 41 | 57 | 3 | 31 | 66 | 61 | 32 | 7 |
|  |  | FIN | 0 | 21 | 79 | 0 | 9 | 91 | 4 | 5 | 91 | 23 | 77 | 0 |
| **TCC HG IN** | 010CR06 | FFPE | 0 | 49 | 51 | 1 | 60 | 39 | 1 | 71 | 28 | 99 | 1 | 0 |
|  |  | FIN | 1 | 51 | 48 | 0 | 62 | 38 | 8 | 80 | 12 | 93 | 7 | 0 |
|  | 013CR06 | FFPE | 0 | 14 | 86 | 0 | 39 | 61 | 0 | 43 | 57 | 97 | 3 | 0 |
|  |  | FIN | 1 | 53 | 46 | 12 | 80 | 8 | 42 | 55 | 3 | 60 | 33 | 7 |
|  | 014CR06 | FFPE | 2 | 60 | 38 | 3 | 91 | 6 | 16 | 81 | 3 | 85 | 13 | 2 |
|  |  | FIN | 32 | 20 | 48 | 7 | 59 | 34 | 6 | 86 | 8 | 90 | 9 | 1 |
|  | 017CR06 | FFPE | 3 | 21 | 76 | 12 | 56 | 32 | 7 | 46 | 37 | 12 | 28 | 60 |
|  |  | FIN | 42 | 34 | 24 | 3 | 19 | 78 | 6 | 23 | 71 | 3 | 18 | 79 |
|  | 019CR06 | FFPE | 3 | 52 | 45 | 3 | 79 | 18 | 6 | 67 | 27 | 58 | 37 | 5 |
|  |  | FIN | 11 | 38 | 51 | 2 | 10 | 88 | 2 | 15 | 83 | 47 | 50 | 3 |
|  | 037CR07 | FFPE | 6 | 27 | 67 | 14 | 58 | 28 | 14 | 55 | 31 | 39 | 37 | 24 |
|  |  | FIN | 0 | 14 | 86 | 0 | 30 | 70 | 0 | 23 | 77 | 20 | 51 | 29 |
|  | 038CR07 | FFPE | 0 | 12 | 88 | 0 | 8 | 92 | 0 | 20 | 80 | 100 | 0 | 0 |
|  |  | FIN | 22 | 30 | 48 | 20 | 42 | 38 | 52 | 42 | 6 | 92 | 6 | 2 |
|  | 040CR07 | FFPE | 0 | 43 | 57 | 2 | 54 | 44 | 2 | 10 | 88 | 7 | 56 | 37 |
|  |  | FIN | 0 | 42 | 58 | 2 | 92 | 6 | 0 | 56 | 44 | 100 | 0 | 0 |
|  | 045CR07 | FFPE | 0 | 34 | 66 | 0 | 51 | 49 | 1 | 45 | 54 | 43 | 30 | 27 |
|  |  | FIN | 16 | 36 | 48 | 34 | 56 | 10 | 70 | 22 | 8 | 98 | 2 | 0 |
