# Supplementary material for: Chromosomal Aberrations in Bladder Cancer: Fresh versus Formalin Fixed Paraffin Embedded Tissue and Targeted FISH versus Wide Microarray-Based CGH Analysis
Source: PLoS One. 2011 Sep 1;6(9):e24237. doi: 10.1371/journal.pone.0024237 (PMC3164716; doi:10.1371/journal.pone.0024237)
Supplement: Table S3 — Copy number alterations (CNA) shared (plus sign) among 10 TCC samples analyzed by array-CGH. NI-TCCs are indicated in italics; IN-TCCs are indicated in bold. For Histology/Grade see Table S1. (DOC) [file pone.0024237.s003.doc]

| Table S3. Copy number alterations (CNA) shared (plus sign) among 10 TCC samples analyzed by array-CGH. NI-TCCs are indicated in italics; IN-TCCs are indicated in bold. For Histology/Grade see Table S1. | | | | | | | | | | |
| --- | --- | --- | --- | --- | --- | --- | --- | --- | --- | --- |
| **GAIN** | ***75CR09*** | ***80CR09*** | ***82CR09*** | ***28CR09*** | **04CR10** | **09CR10** | **10CR10** | **26CR10** | **70CR09** | **81CR09** |
| 1q21.1-q23.3  143699872-161705044 |  |  |  | + |  | + |  | + |  |  |
| 2p25.1  9872881-10505097 |  | + |  | + |  | + |  |  |  |  |
| 2p25.1  11956730-12796508 |  | + |  | + |  | + |  |  |  |  |
| 3p25.2  12328914-12396406 |  |  |  | + |  | + | + |  | + | + |
| 3q25.2  153906953-155170944 |  | + |  | + |  | + |  |  |  |  |
| 3q25.32-q26.1  159876217-162162404 |  |  |  | + | + | + |  |  |  |  |
| 3q26.1  162425422-164314793 |  | + |  | + |  | + |  |  |  |  |
| 5p15.33-p12  204537-45681434 |  | + |  | + |  |  | + |  | + | + |
| 6p24.2-p22.3  11110371-17397320 |  |  |  |  | + | + |  |  |  | + |
| 6p21.1  43589125-44077743 |  |  |  | + |  | + |  |  | + |  |
| 7p22.2-p22.1  4475515-6837609 |  |  |  | + |  | + |  |  | + |  |
| 7p21.1  15397545-19006302 |  |  |  |  | + | + |  |  | + |  |
| 7p15.3  23201784-23537259 |  |  |  | + |  |  |  |  | + |  |
| 7p14.2  39813709-40099186 |  |  |  | + |  |  |  |  | + |  |
| 7q11.21-q11.23  63642906-76078892 |  |  |  | + |  | + |  |  | + |  |
| 7q21.3-q22.2  97323707-104995507 |  |  |  | + |  | + |  |  | + |  |
| 7q32.1-q32.2  128677670-129696226 |  |  |  | + |  | + |  |  | + |  |
| 7q33-q34  137295729-140266986 |  |  |  | + |  | + |  |  | + |  |
| 7q36.1  151407323-152089322 |  |  |  | + |  | + |  |  | + |  |
| 8p11.23-p11.22  39377851-39505456 |  |  |  |  |  | + |  | + |  |  |
| 8q22.3  103489656-104289922 |  |  |  | + |  | + | + |  |  |  |
| 11q13.2-q13.3  68323054-69297218 | + | + |  |  |  | + | + |  |  |  |
| 12q13.11-q14.1  45466239-58591220 |  |  |  | + |  | + |  |  |  |  |
| 13q22.1  72534137-72746128 |  |  |  | + |  | + |  |  |  | + |
| 13q32.2-q32.3  97270228-100084626 |  |  |  |  | + |  |  |  | + |  |
| 14q31.3-q32.11  88632756-89467875 |  | + |  |  |  |  | + |  |  |  |
| 16p13.13  10647794-11795463 |  |  |  | + | + | + | + |  |  | + |
| 16p12.1-p11.2  22221235-32532138 |  |  |  | + |  |  |  |  |  | + |
| 16q11.2-q12.1  45413780-45607724 |  |  |  | + |  | + |  |  |  |  |
| 16q22.2-q22.3  69543004-70813070 |  |  |  | + |  | + |  |  |  |  |
| 17p11.2  24641926-27576519 |  |  |  | + | + | + | + |  |  |  |
| 17q21.1-q25.3  38521264-78444637 |  |  |  | + | + | + | + |  | + |  |
| 18p11.32  467310-796252 |  |  |  | + |  | + |  |  |  |  |
| 19p13.3-p13.13  278073-12616127 |  |  |  |  |  |  | + | + |  |  |
| 19p13.11  18749534-19294814 |  |  |  |  | + | + |  | + |  |  |
| 19q13.11-q13.43  37856935-63485510 |  |  |  | + | + | + | + | + |  |  |
| 20p13  157771-456225 |  | + |  | + |  |  | + | + | + |  |
| 20p11.23  17870221-18440898 |  | + |  | + | + |  | + | + | + |  |
| 20p11.22-p11.21  22093371-26036562 |  | + |  |  |  | + | + | + | + |  |
| 20q11.21-q13.13  28081598-47163674 |  | + |  |  |  | + | + | + | + |  |
| 20q13.13-q13.31  48033501-54958791 |  | + |  | + |  | + | + | + | + |  |
| 20q13.33  61727753-62175139 |  | + |  |  | + |  | + | + | + |  |
| 21q22.11  33581851-34132794 |  |  |  | + |  | + | + |  |  |  |
| 22q12.3  34482413-35495073 |  |  |  |  |  | + |  |  | + |  |
| Xp22.31  6562512-8075294 |  |  |  |  |  | + |  | + |  |  |
| Xp22.11  23836039-24368570 |  |  |  | + |  | + |  |  |  |  |
| Xp11.3  46203719-47229186 |  |  |  | + |  | + |  |  |  |  |
| Xp11.22  53475704-54578960 |  |  |  | + |  | + |  |  |  |  |
| **LOSS** | *75CR09* | *80CR09* | *82CR09* | *28CR09* | **04CR10** | **09CR10** | **10CR10** | **26CR10** | **70CR09** | **81CR09** |
| 1p31.1  77068898-77170790 |  |  |  | + | + |  |  |  |  |  |
| 2p25.3-p16.3  74469-50579433 |  |  |  |  |  |  | + | + |  |  |
| 2p16.3  51236117-51635693 |  | + |  |  |  |  | + |  |  |  |
| 2p16.1  60966923-61033726 |  | + |  |  |  |  | + |  |  |  |
| 2p15  62081310-62298660 |  | + |  |  |  |  | + |  |  |  |
| 2q14.3  123437325-126399586 |  |  |  |  |  |  | + |  |  | + |
| 2q24.2  159948195-160120757 |  | + |  |  |  |  |  | + |  | + |
| 2q35-q37.3  217249378-242464793 |  |  |  | + |  | + |  | + |  | + |
| 3p21.31-p21.1  50653920-52539982 |  |  |  |  |  |  | + | + |  |  |
| 4p14-p13  38697312-42182524 |  |  |  |  |  | + | + |  |  |  |
| 5q11.1-q13.3  50094582-74360979 |  |  |  | + |  | + | + |  |  |  |
| 5q14.3  90316808-90788310 |  |  | + |  |  | + | + |  |  |  |
| 5q31.1  134523261-135051953 |  |  |  |  |  |  | + |  | + |  |
| 6p25.3-p24.3  352263-9584745 |  |  |  | + |  | + |  |  |  |  |
| 6p12.3-p12.2  48489133-52026397 |  |  |  |  |  | + | + |  |  |  |
| 6q21  107127877-107503845 |  |  | + |  |  | + |  |  |  |  |
| 6q21  108783383-109034288 |  | + |  |  |  | + |  |  |  |  |
| 6q22.31  123251233-125408147 |  |  |  |  |  | + |  |  | + |  |
| 8p23.3  211611-432326 |  |  |  |  |  | + |  |  | + |  |
| 8p23.1  6248230-9674346 |  |  |  |  |  | + | + |  |  |  |
| 8p22-p12  13991707-31607745 |  |  |  |  |  | + | + |  |  |  |
| 8p11.23-p11.22  39378051-39505315 |  |  |  |  | + |  | + |  |  |  |
| 9p21.3  21968346-21998655 | + | + |  | + |  | + | + | + |  |  |
| 9q32-q33.2  115832220-122222082 |  | + |  | + |  | + | + | + |  |  |
| 9q34.2-q34.3  135189457-138807183 |  |  |  | + |  |  | + | + |  |  |
| 11p13  31517796-32050223 |  |  |  |  |  |  | + |  |  | + |
| 11q25  130615965-132678521 |  |  |  |  |  | + | + |  | + |  |
| 14q11.2  19364851-19446107 |  |  |  |  | + | + |  |  |  |  |
| 14q24.3-q31.1  77750645-79747731 |  |  |  | + |  | + |  |  | + |  |
| 14q32.31-32.33  101619009-102408085 |  | + |  | + |  | + |  |  |  |  |
| 15q11.2  19427350-19427409 |  |  |  |  |  |  | + | + | + |  |
| 15q26.2  95052889-95403459 |  |  |  |  | + | + |  |  |  |  |
| 16p13.3-p13.2  5996067-6407032 |  |  |  |  | + |  |  |  |  | + |
| 16q12.1-q22.1  46548299-69323131 |  |  |  |  |  | + |  |  |  | + |
| 17p13.3  1649595-1725887 |  | + |  |  |  | + |  |  |  |  |
| 18q22.1-q22.3  61643142-67432063 |  |  |  | + |  |  | + |  |  |  |
| 22q11.1-q11.21  16259597-16597551 |  |  |  |  |  | + | + |  |  |  |
| Yp11.2  7669722-10144057 |  |  |  | + | + |  |  |  | + |  |
