# Supplementary material for: Chromosomal Aberrations in Bladder Cancer: Fresh versus Formalin Fixed Paraffin Embedded Tissue and Targeted FISH versus Wide Microarray-Based CGH Analysis
Source: PLoS One. 2011 Sep 1;6(9):e24237. doi: 10.1371/journal.pone.0024237 (PMC3164716; doi:10.1371/journal.pone.0024237)
Supplement: Table S4 — Gene Ontology. I. Statistically significant (p<0.05) under-representation of gene ontology (GO) categories in HG IN tumors. II. Statistically significant (p<0.05) over-representation of gene ontology (GO) categories in HG IN tumors. III. Statistically significant (p<0.05) under-representation of gene ontology (GO) categories in LG NI tumors. (DOC) [file pone.0024237.s004.doc]

**Table S4.I.** Statistically significant (p<0.05) under-representation of gene ontology (GO) categories in HG IN tumors.

| **GO ID** | **p-Value** | | | | **Description** |
| --- | --- | --- | --- | --- | --- |
|  | **009** | **010** | **026** | **081** |  |
| 0006357 | 3.8x10^-4^ | ns | 3.2x10^-12^ | 6.7x10^-3^ | regulation of transcription from RNA polymerase II promoter |
| 0006366 | 3.2x10^-7^ | ns | 9.8x10^-11^ | 2.1x10^-3^ | transcription from RNA polymerase II promoter |
| 0007049 | 1x10^-9^ | ns | 3.1x10^-7^ | 9.6x10^-3^ | cell cycle |
| 0030154 | 1.4x10^-31^ | 2x10^-3^ | 1.6x10^-3^ | 2.7x10^-3^ | cell differentiation |
| 0043065 | 5x10^-7^ | 2.1x10^-2^ | 5.1x10^-3^ | ns | positive regulation of apoptosis |
| 0043068 | 6.5x10^-7^ | 2.1x10^-2^ | 5.7x10^-3^ | ns | positive regulation of programmed cell death |

ns: not statically significant

**Table S4.II.** Statistically significant (p<0.05) over-representation of gene ontology (GO) categories in HG IN tumors.

| **GO ID** | **p-Value** | | | | | **Description** |
| --- | --- | --- | --- | --- | --- | --- |
|  | **004** | **009** | **010** | **070** | **081** |  |
| 0006357 | 2.2x10^-10^ | 1.2x10^-9^ | 1.5x10^-4^ | 1.5x10^-4^ | 4.8x10^-2^ | regulation of transcription from RNA polymerase II promoter |
| 0006366 | 2.3x10^-11^ | 9.6x10^-16^ | 1.5x10^-4^ | 5.8x10^-4^ | 5.3x10^-3^ | transcription from RNA polymerase II promoter |
| 0006915 | 6.7x10^-10^ | 3.4x10^-4^ | 1.4x10^-2^ | 2.8x10^-7^ | 1.8x10^-2^ | apoptosis |
| 0007049 | 2.2x10^-3^ | 6.8x10^-7^ | 1.9x10^-2^ | 2.8x10^-6^ | 2.9x10^-2^ | cell cycle |
| 0008219 | 2.4x10^-12^ | 2.8x10^-5^ | 1.1x10^-2^ | 1.9x10^-8^ | 1.4x10^-3^ | cell death |
| 0008283 | 5.3x10^-6^ | 2.3x10^-19^ | 1.2x10^-4^ | 5.8x10^-8^ | 9.4x10^-3^ | cell proliferation |
| 0008284 | 1.7x10^-2^ | 4.7x10^-9^ | 2x10^-2^ | 2x10^-2^ | 2.5x10^-2^ | positive regulation of cell proliferation |
| 0012501 | 1.5x10^-10^ | 1.9x10^-4^ | 1.6x10^-2^ | 1.3x10^-7^ | 2.2x10^-2^ | programmed cell death |
| 0016265 | 2.4x10^-12^ | 2.8x10^-5^ | 1.1x10^-2^ | 1.9x10^-8^ | 1.4x10^-3^ | death |
| 0016481 | 8.8x10^-8^ | 8.3x10^-4^ | 8.1x10^-3^ | 4.2x10^-5^ | ns | negative regulation of transcription |
| 0030154 | 2.2x10^-21^ | 1.2x10^-37^ | 3.4x10^-8^ | 3.7x10^-14^ | 1.7x10^-11^ | cell differentiation |
| 0042127 | 3.6x10^-5^ | 2.4x10^-11^ | 4.2x10^-3^ | 1.1x10^-7^ | 9x10^-3^ | regulation of cell proliferation |
| 0042981 | 2.3x10^-7^ | 6.2x10^-3^ | 6.7x10^-3^ | 2.5x10^-6^ | 2.9x10^-2^ | regulation of apoptosis |
| 0043066 | 4.2x10^-4^ | 3.3x10^-3^ | 4.7x10^-2^ | 1.6x10^-2^ | ns | negative regulation of apoptosis |
| 0043067 | 3.8x10^-8^ | 5.4x10^-3^ | 8x10^-3^ | 1x10^-6^ | 2.9x10^-2^ | regulation of programmed cell death |

ns: not statically significant

**Table S4.III.** Statistically significant (p<0.05) under-representation of gene ontology (GO) categories in LG NI tumors.

| **GO ID** | **p-Value** | | | **Description** |
| --- | --- | --- | --- | --- |
|  | **075** | **080** | **082** |  |
| 0006917 | 2x10^-2^ | 4.9x10^-2^ | np | induction of apoptosis |
| 0012502 | 2x10^-2^ | 4.9x10^-2^ | np | induction of programmed cell death |

np: not present
