# Supplementary material for: Chromosomal Aberrations in Bladder Cancer: Fresh versus Formalin Fixed Paraffin Embedded Tissue and Targeted FISH versus Wide Microarray-Based CGH Analysis
Source: PLoS One. 2011 Sep 1;6(9):e24237. doi: 10.1371/journal.pone.0024237 (PMC3164716; doi:10.1371/journal.pone.0024237)
Supplement: Table S5 — Urovysion data. I. Comparison between Urovysion data in two different tumoral areas of the same section II. Comparison between Urovysion data in two different tumoral areas of the same section (DOC) [file pone.0024237.s005.doc]

Table S5.I. Comparison between Urovysion data in two different tumoral areas of the same section

| **SAMPLE** | | **UROVYSION AREA 1** | | | **UROVYSION AREA 2** | | |
| --- | --- | --- | --- | --- | --- | --- | --- |
|  |  | **LOSS%** | **DISOMY%** | **GAIN%** | **LOSS%** | **DISOMY%** | **GAIN%** |
| 075CR09  LG NI | CEP3 | 12 | 59 | 29 | na | na | na |
|  | CEP7 | 2 | 74 | 24 | na | na | na |
|  | CEP17 | 25 | 63 | 12 | na | na | na |
|  | 9p21 | 46 | 49 | 5 | na | na | na |
| 080CR09  LG NI | CEP3* | 4 | 69 | 27 | 4 | 80 | 16 |
|  | CEP7* | 7 | 85 | 8 | 4 | 84 | 12 |
|  | CEP17* | 25 | 72 | 3 | 10 | 82 | 8 |
|  | 9p21* | 100 | 0 | 0 | 100 | 0 | 0 |
| 082CR09  LG NI | CEP3 | 1 | 70 | 29 | na | na | na |
|  | CEP7 | 0 | 86 | 14 | na | na | na |
|  | CEP17 | 7 | 86 | 7 | na | na | na |
|  | 9p21 | 93 | 6 | 1 | na | na | na |
| 028CR09  HG NI | CEP3* | 1 | 43 | 56 | 0 | 47 | 53 |
|  | CEP7 | 3 | 40 | 57 | 0 | 67 | 33 |
|  | CEP17 | 0 | 47 | 53 | 1 | 60 | 39 |
|  | 9p21* | 55 | 41 | 4 | 78 | 21 | 1 |
| 004CR10  HG IN | CEP3* | 20 | 62 | 18 | 14 | 58 | 28 |
|  | CEP7* | 8 | 80 | 12 | 4 | 72 | 24 |
|  | CEP17* | 28 | 68 | 4 | 18 | 61 | 21 |
|  | 9p21 | 70 | 30 | 0 | 28 | 59 | 13 |
| 009CR10  HG IN | CEP3 | 0 | 48 | 52 | 0 | 62 | 38 |
|  | CEP7 | 0 | 44 | 56 | 0 | 56 | 44 |
|  | CEP17* | 0 | 51 | 49 | 0 | 62 | 38 |
|  | 9p21* | 76 | 23 | 1 | 96 | 4 | 0 |
| 010CR10  HG IN | CEP3* | 3 | 66 | 31 | 2 | 49 | 49 |
|  | CEP7* | 0 | 67 | 33 | 1 | 68 | 31 |
|  | CEP17* | 10 | 65 | 25 | 9 | 66 | 25 |
|  | 9p21* | 91 | 8 | 1 | 99 | 1 | 0 |
| 026CR10  HG IN | CEP3* | 0 | 62 | 38 | 0 | 61 | 39 |
|  | CEP7* | 1 | 84 | 15 | 0 | 66 | 34 |
|  | CEP17* | 7 | 74 | 19 | 0 | 75 | 25 |
|  | 9p21 | 32 | 38 | 30 | 97 | 3 | 0 |
| 070CR09  HG IN | CEP3 | 0 | 48 | 52 | 0 | 62 | 38 |
|  | CEP7 | 0 | 56 | 44 | 0 | 42 | 58 |
|  | CEP17 | 10 | 40 | 50 | 6 | 50 | 44 |
|  | 9p21* | 18 | 50 | 32 | 4 | 50 | 46 |
| 081CR09  HG IN | CEP3 | 0 | 42 | 58 | 0 | 59 | 41 |
|  | CEP7 | 0 | 48 | 52 | 2 | 73 | 25 |
|  | CEP17* | 1 | 52 | 47 | 12 | 63 | 25 |
|  | 9p21* | 11 | 47 | 42 | 13 | 54 | 33 |

LOSS: number of signals: 0 and 1; DISOMY: number of signals even (2, 4, 6, 8, 10, 12); GAIN number of signals odd (3, 5, 7, 9, 11). * identifies chromosome probes with **concordant results between the two areas.** Na: not available

**Table S5.II.** Comparison between Urovysion data in two different tumoral areas of the same section

| CASE | PROBE | Student’s *t* test | | |
| --- | --- | --- | --- | --- |
|  |  | Area1 vs Area2 | Area1 vs Total* | Area2 vs Total |
| 080CR09 | CEP3 | 0.008 | 0.157 | 0.096 |
|  | CEP7 | 0.166 | 0.416 | 0.43 |
|  | CEP17 | 0.001 | 0.066 | 0.064 |
|  | 9p21 | 0.318 | 1 | 0.318 |
| 028CR09 | CEP3 | 0.607 | 0.766 | 0.766 |
|  | CEP7 | 0.047 | 0.28 | 0.22 |
|  | CEP17 | 0.267 | 0.522 | 0.52 |
|  | 9p21 | 0.011 | 0.156 | 0.134 |
| 004CR10 | CEP3 | 0.499 | 0.635 | 0.76 |
|  | CEP7 | 0.009 | 0.051 | 0.263 |
|  | CEP17 | 0.018 | 0.093 | 0.297 |
|  | 9p21 | 4.99x10^-10^ | 3.17x10^-5^ | 0.006 |
| 009CR10 | CEP3 | 3.95x10^-8^ | 0.003 | 9.4x10^-4^ |
|  | CEP7 | 2.16x10^-8^ | 0.002 | 9.3x10^-4^ |
|  | CEP17 | 3.7x10^-7^ | 0.006 | 0.002 |
|  | 9p21 | 9.03x10^-13^ | 1.4x10^-4^ | 2.88x10^-5^ |
| 010CR10 | CEP3 | 0.951 | 0.973 | 0.969 |
|  | CEP7 | 0.947 | 0.969 | 0.97 |
|  | CEP17 | 0.628 | 0.787 | 0.77 |
|  | 9p21 | 0.002 | 0.109 | 0.051 |
| 026CR10 | CEP3 | 2.57x10^-6^ | 0.003 | 0.014 |
|  | CEP7 | 5.46x10^-5^ | 0.011 | 0.033 |
|  | CEP17 | 6x10^-6^ | 0.004 | 0.017 |
|  | 9p21 | 4.53x10^-27^ | 1.01x10^-7^ | 5.65x10^-10^ |
| 070CR09 | CEP3 | 0.833 | 0.901 | 0.904 |
|  | CEP7 | 1 | 1 | 1 |
|  | CEP17 | 0.92 | 0.954 | 0.953 |
|  | 9p21 | 0.397 | 0.647 | 0.594 |
| 081CR09 | CEP3 | 0.053 | 0.243 | 0.284 |
|  | CEP7 | 0.131 | 0.375 | 0.391 |
|  | CEP17 | 0.583 | 1 | 0.51 |
|  | 9p21 | 0.083 | 0.297 | 0.334 |
