# Supplementary material for: Chromosomal Aberrations in Bladder Cancer: Fresh versus Formalin Fixed Paraffin Embedded Tissue and Targeted FISH versus Wide Microarray-Based CGH Analysis
Source: PLoS One. 2011 Sep 1;6(9):e24237. doi: 10.1371/journal.pone.0024237 (PMC3164716; doi:10.1371/journal.pone.0024237)
Supplement: Materials and Methods S1 — (DOC) [file pone.0024237.s006.doc]

**Supporting Information S6:** **MATERIAL AND METHODS**

FLUORESCENCE IN SITU HYBRIDIZATION

FISH analysis was performed on both nuclei isolated from FIN and FFPE samples, using UroVysion bladder cancer kit (Vysis, Wiesbaden, Germany), according to the manufacturer’s instructions. For FIN, biopsies were cut up into small pieces with scissors and cultured in RPMI-1640 (Euroclone Spa) supplemented with 20% FCS for 24 hours. Pieces were subjected to hypotonic treatment with 1% sodium citrate at 37°C, then fixed with 3:1 methanol:acetic acid. Single cells were isolated from biopsies with acetic acid solution (acetic acid 60%), then spotted on slides and let dry. They were placed in a pretreatment solution (2X SSC) at 73°C, in a pepsin solution at 37°C, washed in PBS 1X at RT, placed in a post-fixative solution at RT, washed in PBS 1X at RT and dehydrated in ascending alcohol. After the slides were completely air dried, 3 μl of UroVysion probe set was applied to the target area and a cover slip was placed. The slides and probes were codenatured at 73°C for 2 min in a HYbrite denaturation/hybridization system (Abbot/Vysis), then placed in a hybridization chamber overnight at 37°C.

For FFPE, tissue were fixed according to standard procedures; 3 μm tissue sections were baked overnight at 65°C, deparaffinized and dehydrated at room temperature, then immersed in pretreatment solution at 95°C. They were washed in a buffer (Wash Buffer Dako) and then digested with pepsin at 37°C for a variable time (digestion was evaluated with propidium iodide staining). A variable amount between 2 and 5 μl of the UroVysion probe set was added to the tumor scoring area of the slides (defined by H&E staining) and a cover slip was placed. The slides and probes were codenatured at 80°C for 5 min, then placed in a hybridization chamber overnight at 37°C.

After hybridization, for both methods, the unbound probes were removed by a series of washes and the nuclei were counterstained with 4’,6-diamidino-2-phenylindole (DAPI).

At least 100 cells for each preparation were scored and the signals were divided according to loss (number of signals/cell < 2), disomy (number of signals/cell = 2) and gain (number of signals/cell > 2).

For locus 3p25 FISH analysis on FFPE was performed as decribed above, using Poseidon™ Repeat Free™ PPARγ (3p25) Break probe (Kreatech Diagnostics, Amsterdam, Netherlands), a dual-color split probe to detect translocations and amplification at 3p25. Amplification involving the region 3p25 will show 3 or more red-green fusion signals, while two colocalized red/green or yellow signals will identify the normal chromosome(s) 3. The statistical significance of differences between chromosome 3 polisomy (detected by UroVysion CEP3) and 3p25 amplification was evaluated by Student's t test on separate counts of 100 nuclei. Differences were considered as statistically significant with p<0.01.

All digital images were captured using a Leitz microscope (Leica DM 5000B) equipped with a charge coupled device (CCD) camera and analyzed by means of Chromowin software (Tesi Imaging, Milan, Italy).

ARRAY-CGH

For array-CGH analysis, genomic DNA was extracted from fresh biopsies after enzymatic digestion with collagenase H (Roche, Mannheim, Germany) and proteinase K (Roche, Mannheim, Germany) and purified using phenol/chloroform (Carlo Erba, Milan, Italy). The integrity of DNA was confirmed with Nanodrop (Thermo Scientific, Wilmington, DE, USA) and agarose gel electrophoresis. For each hybridization, 1 μg of extracted DNA and 1 μg of sex-matched commercial reference (Promega, Madison, WI, USA) were digested with the restriction enzymes Alu I and RSA I (Promega), and then labeled with Cy-3-dUTP and Cy-5-dUTP, respectively, using the Genome DNA Enzymatic Labeling Kit (Agilent Technologies, Santa Clara, CA, USA). Labeling reactions were assessed by use of the Nanodrop prior to hybridization to the SurePrint G3 Human 60k CGH arrays (Agilent Technologies). Following purification with Microcon Centrifugation Filters, Ultracel YM-30 (Millipore, Billerica, MA, USA), samples were denatured and pre-annealed with 50 μg of human Cot-1 DNA (Invitrogen, Burlington, Ontario, Canada). Hybridization was performed at 65 °C for 40 h with constant rotation. After hybridization, slides were washed according to the manufacturer's instructions and scanned at 2-μm resolution using Agilent microarray scanner and analyzed using Feature Extraction v10.7 and the text files were imported into Agilent Genomic Workbench v5.0 software according to the recommendations of the supplier. The resulting aCGH data were assessed with QC metrics and the Aberration Detection Method 2 (ADM-2) algorithm was used to identify DNA copy number aberrations.

The ADM-2 algorithm (threshold 5.0) identifies all aberrant intervals in a given sample with consistently high or low log ratios based on the statistical score that represents the deviation of the average of the log_2_ ratios from the expected value of zero, in units of standard deviation. The algorithm searches for intervals in which a score quantity based on the average quality weighted log_2_ ratio of the sample and reference channels exceeds a user specified threshold. We applied a filtering option of minimum of 3 aberrant consecutive probes and minimum absolute average log_2_ ratio of 0.3. USCS human genome assembly hg18 was used as a reference and copy number variations (CNV) were identified with a database integrated in the Agilent Genomic Workbench analytic software. Log_2_ ratio lower than -0.3 were classified as losses, those greater than 0.3 as gains and those in between as unchanged. Amplifications were defined as log_2_ ratio values above 1, heterozygous loss as lower than -1 and complete loss as lower than -2. Whole loss or gain of a chromosomal arm as more than 75% of probes with log_2_ ratio less than -0.30 or greater than 0.30. To calculate the estimated percentage of mosaicism we used the formula determined by Cheung SW et al. [28].

GENE ONTOLOGY analysis

To analyse which ontology classes were over and under represented among the genes delineated within gain and loss regions detected by array-CGH, the GOstat software (available at [http://gostat.wehi.edu.au/](http://gostat.wehi.edu.au/cgi-bin/goStat.pl)) was used [29] based on AmiGO (the Gene Ontology database) version 1.8. GOstat software classified genes into different function categories as different GO terms; the output file provides a list of p-value for each GO term, estimating the probability that the observed counts could have occurred by chance.

STATISTICAL ANALYSIS

Cases were described by calculating the proportions of loss, disomy and gain on the total of at least 100 cells, specifically for type of analysis (FFPE and FIN), and probe of UroVysion test (CEP3, CEP7, CEP17 and for the 9p21 region).

A multinomial model accounting for the presence of clustering was used to estimate for each type of tumor and type of analysis, the overall proportion of loss, disomy and gain in each probe with 95% confidence intervals. This multinomial model was also used to compare the overall proportions of loss, disomy and gain detected by the two types of analysis.

A Poisson model based on logarithmic transformation of counts in the presence of clustering was used to estimate the number of signals detected by each type of analysis with 95% confidence intervals. This model enabled also to compare the number of signals across the two types of analysis (FFPE and FIN).
